# Network-level mechanisms underlying effects of transcranial direct current stimulation (tDCS) on visuomotor learning in schizophrenia

**DOI:** 10.1038/s41398-023-02656-3

**Published:** 2023-11-23

**Authors:** Pejman Sehatpour, Johanna Kreither, Javier Lopez-Calderon, Adithya M. Shastry, Heloise M. De Baun, Antigona Martinez, Daniel C. Javitt

**Affiliations:** 1 Division of Experimental Therapeutics, Columbia University Irving Medical Center, New York, NY USA; 2 Schizophrenia Research Division, Nathan Kline Institute for Psychiatric Research, Orangeburg, NY USA; 3 PIA Ciencias Cognitivas, Centro de Investigación en Ciencias Cognitivas, Facultad de Psicología, and Laboratorio de Neurofisiología, Escuela de Medicina, Universidad de Talca, Talca, Chile; 4 Instituto de Matemáticas, Universidad de Talca, Talca, Chile

**Keywords:** Molecular neuroscience, Physiology

## Abstract

Motor learning is a fundamental skill to our daily lives. Dysfunction in motor performance in schizophrenia (Sz) has been associated with poor social and functional outcomes. Transcranial direct current stimulation (tDCS), a non-invasive electrical brain stimulation approach, can influence underlying brain function with potential for improving motor learning in Sz. We used a well-established Serial Reaction Time Task (SRTT) to study motor learning, in combination with simultaneous tDCS and EEG recording, to investigate mechanisms of motor and procedural learning deficits in Sz, and to develop refined non-invasive brain stimulation approaches to improve neurocognitive dysfunction. We recruited 27 individuals with Sz and 21 healthy controls (HC). Individuals performed the SRTT task as they received sham and active tDCS with simultaneous EEG recording. Reaction time (RT), neuropsychological, and measures of global functioning were assessed. SRTT performance was significantly impaired in Sz and showed significant correlations with motor-related and working memory measures as well as global function. Source-space time-frequency decomposition of EEG showed beta-band coherence across supplementary-motor, primary-motor and visual cortex forming a network involved in SRTT performance. Motor-cathodal and visual-cathodal stimulations resulted in significant modulation in coherence particularly across the motor-visual nodes of the network accompanied by significant improvement in motor learning in both controls and patients. Here, we confirm earlier reports of SRTT impairment in Sz and demonstrate significant reversal of the deficits with tDCS. The findings support continued development of tDCS for enhancement of plasticity-based interventions in Sz, as well as source-space EEG analytic approaches for evaluating underlying neural mechanisms.

## Introduction

Schizophrenia (Sz) is a serious mental disorder and a leading cause of long-term disability. Impaired functional outcome is driven largely by impairments in cognitive function that persist despite treatment with best available medications [rev. in [1, 2]]. Non-invasive brain stimulation approaches such as transcranial direct current stimulation (tDCS) are proposed as potential treatments for cognitive dysfunction, especially as a means for enhancing neuroplasticity through enhancement of long-term plasticity (LTP)-like processes [e.g. [3–6]] although optimal approaches need to be developed [e.g., [7–14]]. The Serial Reaction Time Task (SRTT) (also known as the serial finger tapping task, SFTT) has been widely used to study mechanisms of tDCS effects across healthy and neurological populations [rev. in [15–18]] but has been studied in Sz to only a limited degree [e.g. [19, 20]] and without associated biomarkers. Here, we evaluated the sensitivity of the SRTT task to neurocognitive dysfunction in Sz, as well as its sensitivity to tDCS and neurophysiological signature in Sz individuals relative to healthy controls (HC).

**Fig. 1:**
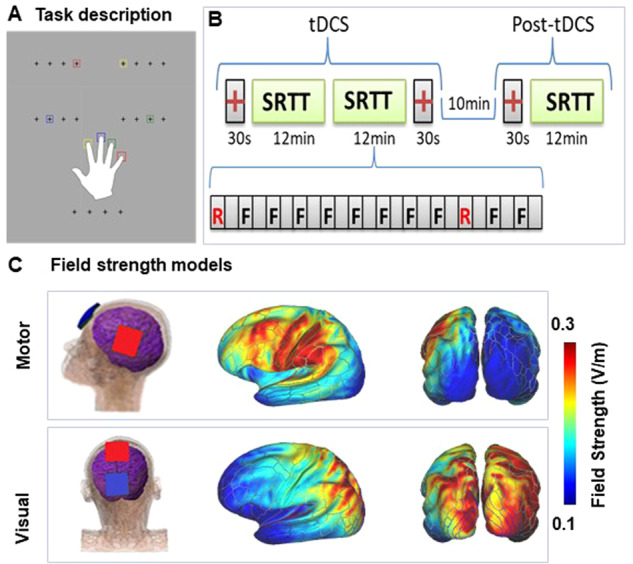
Paradigm and tDCS modeling. **A** Schematic illustration of the task. Participants are instructed to react as quickly to colored squares in one of four positions denoted by crosses that remained on throughout the paradigm by pressing on a spatially and chromatically corresponding button on a keyboard. **B** Task structure. The task used a 5-element repeat sequence that was modeled after previous studies [21, 25, 26, 104]. We used four different SRTT sequences (3, 1, 4, 2, 4), (2, 3, 1, 2, 4), (1, 3, 4, 2, 3), (4, 2, 1, 3, 2), pseudo randomly assigned to one of the four stimulation conditions i.e., Sham, Motor-anodal, Motor-cathodal and Visual-cathodal, per individual, such that no one received the same sequence twice. Two blocks of SRTT, 12 min each, were administered during tDCS/EEG. **C** tDCS Field Strength Mapping. Pad placements for the Motor-cathodal and Motor-anodal conditions followed the M1-SO (left primary motor-right supraorbital) scalp positions used in prior tDCS SRTT studies. For Visual cortex stimulation, the anode pad was placed over the vertex (Cz) and the cathode pad was placed on the scalp area (POz) overlaying the cortical dorsal visual area [76]. For sham stimulation, the pads were placed in the same positions as for motor stimulation; however, the stimulator only delivered 30 seconds of ramp up and down. The montage resulted in predominant current flow in premotor and somatomotor regions during motor cortex stimulation and dorsal visual and superior parietal regions during visual cortex stimulation [25].

In the SRTT, a fixed sequence of visual targets is presented repeatedly on a computer screen (Fig. 1A). When the sequence is random, the mean reaction time (RT) across trials remains relatively constant. In contrast, when the sequence repeats, individuals show a progressive reduction in RT over repeat trials even if they are not consciously aware of the sequence, reflecting implicit motor learning. The SRTT has been widely employed as an instrument to measure tDCS effects in part because of the ready accessibility of motor cortex to stimulation [rev. in [15]]. For example, tDCS stimulation over the primary motor cortex (M1) has been shown to increase learning when applied during the task [e.g. [21, 22]], whereas tDCS applied over parietal cortex enhances later stages of consolidation [23].

In the SRTT, the progressive reduction in RT during stimulus repetition primarily reflects a shift in individual responses from a slow, “reactive” mode (equivalent to a choice-reaction time task) in which the stimulus is needed to determine both where and when to press; to a fast “proactive” mode (equivalent to a simple reaction time task) in which the location of the stimulus has been predicted in advance and the stimulus indicates only when to press [24, 25]. In HC, we have previously demonstrated that tDCS applied over either motor or visual cortex increases the shift from slow to fast responses along with changes in both EEG coherence and fMRI functional connectivity between visual and motor regions [25, 26]. Here, we evaluate the degree to which similar effects can be achieved in Sz.

In Sz, cognitive impairments are assessed primarily using paper-and-pencil batteries such as the MATRICS consensus cognitive battery (MCCB) [27]. While effective, such tasks are poorly suited to analyzing either the neural mechanisms underlying cognitive impairments or the potential mechanisms by which tDCS could reverse underlying dysfunction. An advantage of the SRTT is that the underlying cortical circuitry has been extensively evaluated and is known to depend upon the interaction of components of the motor cortex and the prefrontal supplementary motor area (SMA) region [5, 16, 28–32] with primary visual cortex [33] and the dorsal stream visual “action” system [34]. Here, in order to interrelate SRTT performance to more traditional neurocognitive domains in Sz, we collected parallel data using both the MCCB and the Purdue Pegboard Test [35, 36], which serves as a test of both procedural learning and motor dysfunction across neuropsychiatric disorders.

At the electrophysiological level, interaction among regions involved in SRTT performance is indexed by coherent event-related desynchronization (ERD) of ongoing beta-frequency (12–24 Hz) rhythms within the extended motor network [e.g., [37–44]]. Task-dependent modulation of motor activity within the extended visuomotor networks, including in the SRTT, is reflected in alterations in coherence within the β (12-24 Hz) frequency range [29, 45, 46], as well as in fMRI functional connectivity between regions [25, 47]. Nevertheless, optimal approaches for applying and guiding tDCS using neurophysiological brain measurements remain to be determined. To date, repeated tDCS targeted at specific brain regions has shown promise for treatment of specific symptomatic features, such as persistent auditory hallucinations [e.g. [48, 49]] or lack of insight [50]. Nevertheless, studies seeking to use tDCS to enhance neuroplasticity in Sz have shown mixed success. For example, while some studies have found significant tDCS enhancement of LTP-like activity during repeated visual stimulation [51], others have reported negative results and have emphasized the need for further studies [52].

Against this background, the goal of the present study was three-fold. First, we evaluated the degree to which the SRTT may be useful in assessing neural mechanisms underlying specific aspects of neurocognitive dysfunction in Sz. Second, we evaluated the relative effectiveness of active vs. sham tDCS over motor and visual cortex in Sz relative to HC. Finally, we evaluated the degree to which β-coherence measures could be used to assess tDCS effects across populations. Based upon prior findings of impaired dorsal stream visual function in Sz and its effects on higher level processing [53–59], we hypothesized that impaired SRTT performance in Sz would be related in part to impaired interaction of visual cortex with other nodes of the visuomotor system as well as local dysfunction within motor and premotor regions, and that beneficial effects would thus be obtained from tDCS applied over both motor and visual sensory regions.

## Methods

### Participants

Participants included 21 healthy controls (HC) aged 18–50 and 27 individuals with schizophrenia (Sz), aged 18–50 (Table 1). Patients were recruited from inpatient and outpatient facilities associated with the Nathan Kline Institute for Psychiatric Research. Controls were recruited from Nathan Kline Institute’s database of healthy volunteers. All participants provided written informed consent, and the procedures were approved by the Nathan Kline Institute/Rockland Psychiatric Center Institutional Review Board and ethics committee. All participants reported normal or corrected-to-normal vision. All were right-handed. Symptom ratings were obtained using the Positive and Negative Symptom Scale (PANSS). Neuropsychological assessment included the Quick IQ Test [60], MATRICS consensus cognitive battery (MCCB) neurocognitive domains, and the Purdue Pegboard task [61, 62]. Global function was assessed using the Independent Living Scale (ILS) [63] and Generalized assessment of function (GAF). Data from 3 of the HC were included in a prior report [25].

**Table 1:** Demographics.

	Control (*n* = 21)	Schizophrenia (*n* = 27)			
	Mean	SD	Mean	SD	*P*-Value
Gender (% female)	8	38.1	6	23.1	0.82
Age	34.7	10.7	35.0	10.0	0.92
Participant SES	46.4	10.1	26.1	7.0	**<0.001**
Parental SES	44.1	14.0	39.8	10.9	0.25
Quick IQ [60]	102.8	7.3	97.5	9.5	0.053
Purdue Pegboard (Trial 1)	33.9	6.7	20.7	7.8	**<0.001**
Purdue Pegboard (Trial 2)	35.1	1.5	21.4	8.0	**<0.001**
Purdue Pegboard (Trial 3)	36.6	7.8	22.4	8.35	**<0.001**
MCCB (SoP)	48.7	8.6	34.8	11.7	**<0.001**
MCCB (AttVig)	49.7	8.9	36.9	11.5	**<0.001**
MCCB (WM)	49.2	8.3	38.1	13.0	**0.002**
MCCB (VerL)	45.4	8.0	36.7	8.4	**0.001**
MCCB (VisL)	40.4	12.3	33.4	15.8	**0.120**
MCCB (RPS)	50.0	12.4	38.8	9.1	**0.002**
PANSS Positive	–	–	13.1	3.5	–
PANSS Negative	–	–	15.3	4.9	–
PANSS Cognitive	–	–	10.9	3.4	–
Medication dose (CPZequiv)	–	–	976.3	864.1	–
Typical/Atypical/Both (# individuals)			4/15/6		–
Anticholinergic (# individuals)			10		
Illness duration (yrs)	–	–	13.1	7.6	
GAF			49.5	12.8	

*SES* Socioeconomic status (Edinburgh scale), *MCCB* MATRICS Consensus Cognitive Battery, *SoP* Speed of processing, *Attvig* Attention/Vigilance, *WM* Working memory, *VerL* Verbal Learning, *VisL* Visual Learning, *RPS* Reasoning and Problem Solving, *PANSS* Positive and Negative Symptom Scale, *CPZe* chlorpromazine equivalents, *GAF* General Assessment of Function. Typical antipsychotics included fluphenazine (9), haloperidol (3),), perphenazine (1), chlorpromazine (1); atypicals included risperidone (8), clozapine (8), olanzapine (7), aripiprazole (3), quetiapine (2), lurasidone (1), paliperidone (1). Anticholinergics included benztropine (9), diphenhydramine (1). One Sz participant was also taking lithium.

Bold values indicates statistical significant *P* values.

### Stimuli and experimental design

As previously described [25], stimuli consisted of colored squares that appeared in one of four positions, designated by crosses that collectively subtended ±1.4° visual angle from the center of the screen. On each trial, participants pressed one of four visually cued color-coded keys on a standard computer keyboard with the fingers of their right hand as quickly and accurately as possible following presentation of a cue (Fig. 1A). Each block consisted of 12 self-paced 1-min runs, with random runs at positions 1 and 10 of the sequence (e.g. [21]). A single block was repeated 10-min post-tDCS (Fig. 1B).

### Behavioral data analysis

For baseline analyses, RT data from random and fixed runs were log-transformed and averaged across trials within a block. Mean values were compared across groups using repeated measures ANOVA with within-individual factor of Block and between-individual factor of Group status. Partial correlations controlling for group status were used to assess the relationship between RT and clinical data across participants. To assess effects of tDCS, a mixed-model regression was performed across runs, with run as a co-variate and Group membership and tDCS condition as factors. For single-trial analyses, single-trial log-RT distributions were compared across conditions using single vs. dual-Gaussian models using GraphPad 9.0 non-linear curve fitting functions, as described previously [25] (see Supplementary methods).

### tDCS

tDCS was applied by a saline-soaked pair of surface sponge pads (3 × 3 cm) using the battery-driven, NeuroConn DC-Stimulator MR (NeuroConn, Ilmenau, Germany). During the ERP section of the study, the participants received four stimulation conditions (Sham, Motor-cathodal, Visual-cathodal, Motor-anodal) using a constant current of 2-mA intensity applied for 30 minutes during the task performance (Fig. 1B). Each stimulation condition was administered on a separate day at least 36-hours apart for each participant in counterbalanced order, based on prior studies showing limited effects beyond 24-hours [64, 65]. The study was double-blind with a cross -over design. Data were unblinded only following completion of all within-subject EEG analyses. Finite-element modeling of electric field strength was performed on the MNI-152 head (6^th^ generation, non-linear-T1-weighted), using the ROAST [66] toolbox in MATLAB. Electrical field strength outputted by ROAST as NIfTI volume was then mapped onto the standard averaged MNI surface [25] (Fig. 1C).

### tDCS discomfort

We used the Wong-Baker Faces Pain Scale [67] to measure discomfort caused by tDCS application after each session. This scale consists of 6 faces with face 0 indicating a happy face denoting “No Hurt” and face 5 indicating a crying face denoting “Hurts Worst”. This scale illustrates physical pain and is easy to understand. We also asked the participants to verbalize what type of hurt they felt if any including itching, burning, pain, or other.

### EEG data acquisition

Continuous EEG along with digital timing pulses representing key presses was acquired through Brainvision Brainamp MR Plus amplifier system using 32 scalp active electrodes, impedances <5 kΩ, referenced to the FCz electrode, bandpass filtered from 0.05 to 100 Hz, and digitized at 500 Hz. Data were re-referenced to average-reference and analyzed offline using BESA Research, version 6 (Brain Electric Source Analysis, BESA GmbH), EEGLAB [68], ERBLAB [69] and Matlab software, version 2017a (MathWorks).

Data were epoched from −400 to +200 ms relative to key motor response and were subjected to both automated (±70 $\mu$V at all scalp sites) and manual artifact rejection. Electrode positions that were removed to accommodate the tDCS pads were interpolated using Spherical Spline Interpolation [70]. Epochs were subjected to time-frequency transformation using complex demodulation [71, 72] for frequencies of 4–50 Hz. Frequencies were sampled in 2-Hz steps with the sampling rate of 500 Hz (2 ms).

As in our prior studies [25, 26], analyses focused on the –200-0 ms pre-motor interval, relative to the prior 200 ms (-400 to –200 ms baseline). While the entire range of 4–50 Hz was interrogated significant modulations in the pre-motor interval versus baseline were only observed in the β frequency range in this paradigm. β-ERD values were calculated using temporal spectral evolution (TSE) defined as the relative power change at a time-frequency bin (–200 to 0 ms premotor interval and frequency range of 12–24 Hz) compared with the mean power over the baseline epoch (–400 to –200 ms premotor interval for that frequency [37, 72]. Intracranial sources of beta-activity were assessed using a Beamformer approach, to define the canonical brain regions involved in the task performance. These regions were then used to derive coherence across the cortical regions as described previously [25, 73–75]. (see Supplementary Methods).

### Statistics

The sample sizes were selected to provide power = 0.8 to detect large between-group differences (*d* = 0.8), moderate (*f* = 0.25) within-group effects of tDCS across conditions and large (*r* = 0.5) within-group correlations in Sz. Data were evaluated for normality prior to statistical evaluation and homogeneity of variance across groups. All statistical tests were two-tailed, with preset alpha level for significant of *p* < 0.05.

## Results

Initial analyses focused on between-group SRTT performance during the sham condition between Sz and HC, and its relationship to underlying neurophysiological (β-ERD) responses. Subsequent analyses focused on the magnitude and mechanism of tDCS effects across groups.

### Baseline performance

#### Mean RT analyses

**Fig. 2:**
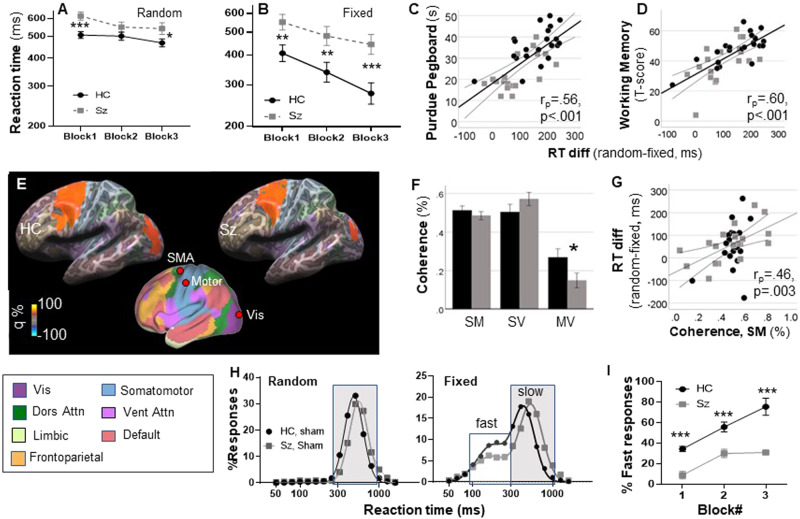
Between group comparison during sham stimulation. Reaction time (RT) by block for the random (**A**) and fixed (**B**) conditions. **C***,* **D** Correlations with indicated neuropsychological tests. **E** Mean source space solutions for premotor β-event-related desynchronization (ERD) responses, showing the location of the supplemental motor area (SMA), motor, and visual (Vis) sources. q values represent normalized power within the time-frequency bin of interest. **F** Coherence values for SMA-motor (SM), SMA-visual (SV) and motor-visual (MV) sources. **G** Correlation between basal coherence in the SM connection and RT differences across groups. **H** Single-trial RT histograms for HC and Sz participants in the random and fixed condition, pooled across blocks for calculation of mean RT values. **I** Percent fast responses in the fixed condition as a function of Group and Block. *p < 0.05, **p < 0.01, ***p < 0.001.

In the random condition (Fig. 2A), there was a linear effect of Block (F_1,46_ = 18.5, *p* < 0.001) reflecting gradual improvement across groups. There was also a significant main effect of Group (F_1,46_ = 5.14, *p* = 0.03) reflecting longer RTs in the Sz group across blocks. As expected, the linear Block X Group effect was not significant (F_1,46_ = 1.00, *p* = 0.3). In the fixed condition (Fig. 2B), both the linear effect of Block (F_1,46_ = 85.4, *p* < 0.001) and the main effect of Group (F_1,46_ = 8.74, *p* = 0.005) were highly significant. In addition, there was a significant linear Group X Block interaction (F_1,46_ = 7.14, *p* = 0.01) reflecting a differential slope across groups.

In a combined analysis across the random and fixed condition, there was a significant main effect of group (F_1,46_ = 7.66, *p* = 0.008), a significant task X group interaction (F_1,46_ = 10.5, *p* = 0.002) reflecting the greater deficit observed in the fixed vs. random version of the task, and a highly significant 3-way linear task X block X group interaction (F_1,46_ = 23.8, *p* < 001), demonstrating that the differential change in slope across the two-tasks was statistically reliable across groups.

#### Comparison with traditional neurocognitive measures

Sz was also associated with increased time to complete the Perdue Pegboard Task, along with reductions in neuropsychological performance across MCCB domains (Table 1). Relative increases in RT for the fixed vs. random version of the task correlated strongly with reduction in performance in the Assembly Trial of the Perdue Pegboard (r_*p*_ = 0.56, *p* < 0.001, Fig. 2C) as well as the Working Memory T-score of the MCCB (r_*p*_ = 0.60, *p* < 0.001) (Fig. 2D), with weaker correlations to Speed of Processing, Attention/Vigilance, and Visual Learning and Reasoning/Problem Solving (all *p* < 0.05). When these measures were entered into a simultaneous regression, the Purdue Pegboard (r_*p*_ = 0.36, *p* = 0.033) and MCCB Working Memory (r_*p*_ = 0.43, *p* = 0.01) were independently significant and accounted for 57.3% of the variance in SRTT performance (*p* = 0.009). Once these variables were entered into the regression, no other measures contributed significantly.

When analyses were conducted separately by group, the correlations with Purdue Pegboard were significant for Sz (*r* = 0.58, *p* = 0.006) and HC (*r* = 0.46, *p* = 0.039) separately, as were the correlations with working memory (Sz: r = 0.53, *p* = 0.011; HC: *r* = 0.51, *p* = 0.021). In Sz, performance on the SRTT did not correlate significantly with medication dose, illness duration, or symptom severity (all *p* > 0.2), although it did correlate with higher level measures including general function (GAF, *r* = 0.50, *p* = 0.028), function capacity (ILS, *r* = 0.50, *p* = 0.027) and participant (*r* = –0.49, *p* = 0.027), but not parental (*r* = 0.18, *p* = 0.49), socioeconomic status.

#### Neurophysiology

In order to evaluate neurophysiological bases of the behavioral SRTT deficits in Sz, coherence analyses were performed on the pre-movement β-activity (within the time-frequency range defined above). As reported previously [25, 26], significant β-ERD was observed within the premotor, motor, and visual sensory regions, which mapped to the canonical dorsal attention, somatomotor and visual networks [76], respectively (Fig. 2E).

The magnitude of the β-ERD did not differ significantly between groups under the sham condition. In contrast, there was a highly significant Group X Connection interaction across connections (F_2,38_ = 5.29, *p* = 0.009) in coherence, reflecting a significant reduction in coherence in the Motor-Visual pathway in the Sz versus HC group during the pre-movement period (F_1,39_ = 4.69, *p* = 0.037) (Fig. 2F). Across groups, the initial difference in RT in the fixed vs. random task correlated significantly with the baseline SMA-Motor cortex coherence (r_*p*_ = 0.46, *p* = 0.003) (Fig. 2G).

#### Single trial analyses

In single-trial analyses, as in our previous study [46], data were best fit by a single Gaussian function during the random condition. Mean log-RT was significantly longer in the Sz (2.74 ± .004 log-ms; 550 ms) vs. HC (2.68 ± .002 log-ms; 479 ms) group (F_1,26_ = 220.5, *p* < 0.0001). For the fixed condition across blocks, data fit better to a 2-Gaussian model for both the HC (F_3,10_ = 152.1, *p* < .0001) and Sz (F_3,10_ = 70.0, *p* < 0.0001) groups, with separate populations of fast (“proactive”) and slow (“reactive”) responses (Fig. 2H). As with slow responses, the mean RT of the fast response mode was also ~50 ms longer in the SZ (2.44 ± .04 log-ms; 275.4 ms) than HC (2.36 ± 0.02 log-ms; 229.1 ms) group. In both groups, the percentage of fast responses increased progressively across blocks. Across all blocks, the percentage of fast responses was substantially lower for Sz than HC (F_1,24_ = 40.2 *p* < 0.0001) (Fig. 2I).

### Effects of tDCS

Effects of tDCS were assessed using both traditional (mean RT) and single-trial approaches. For the mean RT analyses, in order to control for the general psychomotor slowing in SZ, values from the fixed runs were normalized to those in the random runs and expressed as % reduction in RT relative to the mean random RT. As no effects of tDCS were observed for the random condition, a common normalization value was used across all conditions.

#### Mean RT

**Fig. 3:**
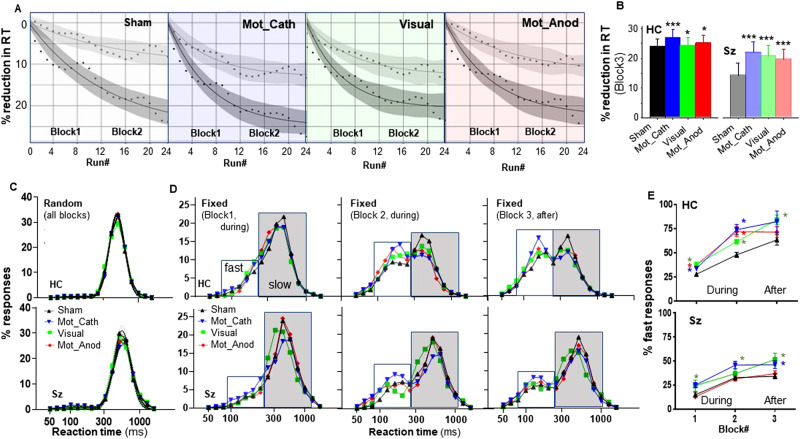
Effect of tDCS on SRTT behavior. **A** Reduction in reaction time (RT) across runs for the Sham, Motor-Cathodal (Mot_Cath), Visual Cathodal, and Motor Anodal (Mot_Anod) condition. Runs 1-12 corresponding to block 1 and runs 13-24 corresponding to block 2. In all cases, curves fit well to an exponential improvement function. **B** Effects of tDCS on mean RT across runs in block 3 by group. **C** Superimposed RT histograms across blocks and stimulation condition in the Random task. Note unimodal response profiles. **D** RT histograms for the fixed condition by Group, Block and Condition. Note bimodal RT distributions, with shift from slow to fast response mode in both groups. **E** Percent fast responses by Group and Condition. Data are mean ± sem. Data are mean ± sem. *p < 0.05 across conditions for condition indicated by color vs. sham.

Mean RT was analyzed using both ANOVA by block and stimulation type (Table 2) and using an MMRM with factors of Group and tDCS Condition, and with Run as a covariate. During stimulation, there was a significant main effect of Group (F_1,46.2_ = 5.59, *p* = 0.022) as well as a highly significant Group X Run interaction (F_1,202684_ = 98.1, *p* < 0.001), reflecting reduced improvement over time in Sz versus HC participants. The main effect of Condition (F_3,202685_ = 90.2, *p* < 0.001) and the Condition X Group interaction (F_3,202685_ = 25.6, *p* < 0.001) were also strongly significant. Across groups, all tDCS conditions were significantly beneficial, with order Mot_Cath>Visual>Mot_Anod>Sham (Fig. 3A).

In order to evaluate the degree to which improvement was maintained following stimulation, a separate analysis was performed for block 3 (Fig. 3B). As for the earlier blocks, there were significant main effects for Group (F_1,46_ = 15.2, *p* < 0.001) and tDCS Condition (F_3,117847_ = 218.5, *p* < 0.001). For both groups, significant enhancement of plasticity was observed for all tDCS conditions. Although the order of effectiveness was similar for the two groups, the relative degree of improvement was larger for the Sz than HC as shown by a significant Group X Condition interaction (F_3,117865_ = 46.3, *p* < 0.001).

#### Trial by trial analyses

In the trial-by-trial analyses, tDCS was again without effect on performance in the random condition in either group (Fig. 3C). In the fixed condition, bimodal fits were observed in all conditions, with the expected progressive shift from slow to fast responses across blocks (Fig. 3D). Consistent with the mean RT results, tDCS significantly increased the percentage of fast vs. slow responses for both HC and Sz across blocks, with largest effect for Motor-cathodal and Visual-cathodal stimulation (Fig. 3E). Whereas Motor-anodal stimulation produced significant effects during stimulation in HC participants, no significant effects were observed in Sz. In Sz, both Motor-cathodal and Visual-cathodal stimulation produced effects that persisted following stimulation.

#### Order effects

There was no significant effect of order (F_3,125_ = 0.473, *p* = 0.7) or order X group (F_3,125_ = 0.482, *p* = 0.69), or order X condition interaction (F_9,125_ = 0.698, *p* = 0.7). There were also no significant order effects within each group.

#### Neurophysiology

The magnitude of the β-ERD was assessed by univariate ANOVA with factors of Group, tDCS Condition and region. The results indicated only a main effect of group (F_1,1032_ = 26.15, *p* = 0.001). There were no other significant main or interaction effects. No significant correlations between magnitude of the β-ERD and RT were observed at any of the regions of interest even after controlling for group and stimulation condition.

**Fig. 4:**
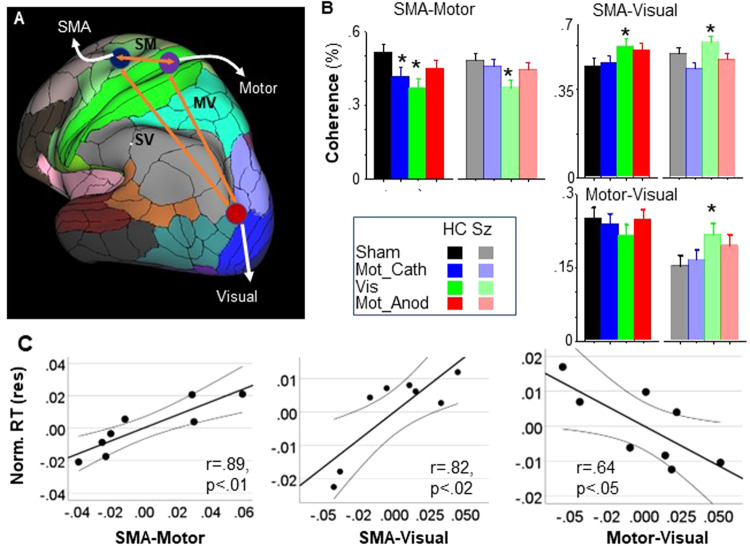
Source Coherence. **A** Schematic of interconnections analyzed using Beamformer coherence approaches. **B** Cluster permutation analysis of coherence measures under indicated tDCS conditions for the SMA-Motor, SMA-visual and Motor-Visual connections. Data are mean ± sem. **C** Scatter plots (with 95% confidence interval) of normalized RT by indicated coherence values across conditions *p < 0.05.

tDCS effects on between-region coherence levels were assessed by univariate ANOVA with factors of tDCS Condition and Group. tDCS significantly modulated coherence in both the SMA-Motor (F_3,114_ = 6.10, *p* < 0.001) and SMA-Visual (F_3,112_ = 4.08, *p* = 0.009) connections (Fig. 4A, B). In both cases, the modulation was most robust for stimulation over visual cortex. For Motor-Visual connectivity, there was a significant Group X Condition interaction across the Sham- and Visual-stimulation conditions, reflecting a non-significant reduction in coherence in the HC group vs. a significant increase in Sz (F_1,38_ = 7.00, *p* = 0.012).

In order to evaluate the interrelationship between behavior and neurophysiological measures, we conducted regression analyses at both the individual subject and group levels. In a mixed-model regression of normalized RT by subject and group, both SMA-visual (F_1,443_ = 4.022 and Motor-visual (F_1,443_ = 4.66, *p* = 0.031) significantly predicted normalized RT across groups. In addition, the two-way group X Motor-visual coherence (F_1,443_ = 4.18, *p* = 0.041) and SMA-visual X Motor-visual coherence (F_1,443_ = 5.19, *p* = 0.023) effects and the 3-way group X SMA-visual X Motor-visual coherence effect (F_1,443_ = 5.51, *p* = 0.019) were all significant. Once these covariances were considered the between-group difference in normalized RT was no longer significant (F_1,443_ = 0.84, *p* = 0.36). When analyses were repeated at the group mean level across condition, there was a highly significant overall relationship between normalized RT and the coherence pattern (F_4,3_ = 38.2, *p* = 0.007), with oppositive patterns in relationship to SMA-Motor and SMA-Visual vs. Motor-Visual cortex coherence (Fig. 4C).

#### Discomfort

All subjects tolerated the tDCS/EEG. None of the participants reported a score higher than 2 on the scale of 0-5 (mean = 1.02, SD ± 0.2). No significant group differences were found (F_1,175_ = 2.72, *p* = 0.101), no significant effect of tDCS condition (F_3,175_ = 2.04, *p* = 0.111) and no interaction effect. The subjective feeling of discomfort was primarily reported as itching (65%), tingling (24%) and burning (11%). No adverse events occurred during this study.

**Table 2:** Effect of tDCS on mean RT by block and stimulation type.

Ctl	Overall		Sham vs. Mot_Cath		Sham vs. Visual		Sham vs. Mot_Anod	
	F (1,48)	*p*	F (1,24)	*p*	F (1,24)	*p*	F (1,24)	*p*
Block1	6.37	0.001	7.17	0.013	28.3	<.0001	11.8	0.0022
Block 2	11.4	<.0001	23.6	<.0001	17	0.0004	20.68	0.0001
Block3	54.6	<.0001	1.51	0.23	5.76	0.025	1.07	0.31
Sz	**Overall**		**Sham v. Mot_Cath**		**Sham v Visual**		**Sham v Mot_Anod**	
	**F**	***p***	**F**	***p***	**F**	***p***	**F**	***p***
Block1	3.411	0.025	7.36	0.012	2.4	0.13	0.46	0.51
Block 2	2.943	0.042	9.43	0.005	0.55	0.47	0.13	0.73
Block3	3.95	0.014	7.01	0.014	7.21	0.013	0.51	0.45

## Discussion

Sz is associated not only with persistent cognitive impairments, but also with impairments in cortical plasticity that limit the ability of individuals to improve performance with practice. tDCS enhances plasticity and learning capacity in healthy individuals [e.g. [4, 6, 23, 51]], but studies in Sz to date have yielded mixed results [e.g. [51, 52, 77]]. Here, we evaluated tDCS effects on implicit visuomotor learning using the SRTT combined with neurophysiological indices of interactions within a distributed premotor, motor and visual circuit.

Principal findings are three-fold. First, we confirm earlier reports of SRTT impairment in Sz [19], and demonstrate that the impairment as expected reflects a reduced shift from reactive to proactive responses. Second, we demonstrate that as in HC, tDCS applied over both motor and visual regions significantly enhances motor learning. Finally, we interrelate these with alterations in β-coherence between nodes of the underlying visuomotor circuit. Overall, these findings support a continued focus on development of tDCS for enhancement of plasticity-based interventions in Sz, as well as EEG biomarker-based approaches for evaluating underlying neural mechanisms.

### Visuomotor/procedural learning deficits in Sz

Cognitive dysfunction extends across a range of cognitive domains. However, over recent years there has been increasing realization that motor aspects of Sz are both important and understudied [78, 79]. Nevertheless, optimal tests for the investigation of neural mechanisms underlying motor dysfunction need to be identified. Here, we benchmarked the SRTT against the Purdue Pegboard Test.

In Sz, deficits in manual dexterity measured using the Purdue Pegboard predate illness onset and are among the strongest predictors of conversion to Sz among prodromal individuals [80]. In established Sz, reduced Purdue Pegboard performance is associated with diffuse white matter disorganization [81]. Our study supports distributed network models of SRTT dysfunction in Sz, with particular emphasis on contributions of visuomotor connectivity [25]. These findings are also consistent with prior studies demonstrating that impaired visual input into prefrontal cortex contributes to fragmented object recognition deficits in Sz [53, 82], which can also be disrupted in healthy individuals using TMS applied over the dorsal visual stream [54].

Deficits in SRTT performance are observed not only in Sz but also in Parkinson’s disease (PD), specific language impairment (SLI), and dyslexia [19]. The shared deficit with PD suggests that SRTT dysfunction in Sz may be related to known dopaminergic disturbances. However, the shared deficit with SLI and dyslexia argues that alternative mechanisms may also be critical and of specific relevance to Sz. For example, deficits in dorsal-stream visual performance in Sz contribute to low-level reading disturbances computationally similar to those observed in dyslexia and SLI [83–85].

Here, we observed two components of slowing in the SRTT. First, both proactive (fast) and reactive (slow) responses were ~50 ms slower across the random and fixed conditions. However, in the fixed condition, the majority of the deficit related to the reduction in the shift from slow to fast responses. How these patterns relate to those observed in other disorders remains to be determined. In our study, mean RT did not correlate with medication dose for either the random or fixed condition, arguing against medication-induced dopaminergic blockade as an underlying mechanism.

### tDCS

Our demonstration of tDCS effects on SRTT performance in healthy individuals using the traditional mean RT approach is consistent with extensive prior literature [e.g. [15, 25, 26]]. In addition, our single trial analysis confirmed that reductions in mean RT across runs correspond to motor learning, as reflected in a shift from slow to fast responses, rather than a change in mean RT of either response type independently.

In both mean RT and single-trial analyses, Motor-cathodal and Visual-cathodal stimulation proved most effective. Moreover, the degree of improvement in motor learning in Sz was significantly larger than in HC. Of note, although tDCS reduced the difference between the HC and Sz groups, it did not restore performance in Sz to control levels. In the present study, in order to remain compatible with prior literature, we used both group-mean field strength mapping and a low-density montage. Future studies with high-definition approaches [26], personalized mapping [86] and repeat administration may yield even further benefit.

### Neurophysiological outcome

As recently reviewed [87], EEG measures provide potential biomarkers of tDCS effect, but relatively few studies have been conducted to date. Both β-ERD [e.g. [37]] and coherence measures among scalp electrodes were considered promising approaches. Here, we further refine the coherence approach by conducting the analyses within source-space using a Beamformer approach that we have previously validated relative to underlying fMRI connectivity patterns [25].

Here, we replicate the β-ERD source distribution in a new cohort of HC and Sz individuals, while also providing novel evidence for impaired Motor-Visual connectivity under non-stimulation conditions in Sz (Fig. 2F), and its potential amelioration especially by tDCS applied over visual cortex (Fig. 4B), which correlated with alterations in coherence levels across subjects and group (Fig. 4C). Our finding of impaired β-coherence is consistent with a more general literature showing dysregulation of synchronous neural oscillations as a mechanism in pathophysiology of brain disorders [88] and in particular, abnormal β-frequency synchronous oscillations across cortical networks in Sz [89].

Even though β-coherence was significantly affected by both Group and Condition, β-ERD amplitude was not affected by stimulation condition, consistent with other recent literature [90–92]. These findings are consistent with the increasing appreciation of Sz as a disorder of functional connectivity [e.g. [53, 93–95]]. In contrast, the β-ERD has been shown to be reduced in primary motor disorders such as stroke, amyotrophic lateral sclerosis, dystonia and PD [90].

Thus, while disorders such as Sz and PD have shared SRTT deficits, underlying neural mechanisms may be significantly different. For example, a recent study did not find a significant effect on acute tDCS over motor cortex on either sequence learning or hemodynamic response in PD [96]. The SRTT is well-suited to EEG-based analysis because of the large number of trials and the ability to “back-average” from the motor response. The present findings suggest that simultaneous EEG recording, especially when combined with network-level analysis, may assist in differentiating underlying neural mechanisms across disorders.

### Limitations

Although we show significant correlated effects of tDCS on behavior and network coherence, several limitations of the study should be considered. First, we used a low-density, non-personalized montage. Especially for the Visual cortex stimulation, current flow was not optimized to the region of greatest network-level impairment. In a more recent study in HC, we found that high-definition tDCS over visual cortex led to significantly greater effect than with the present montage [26]. Future studies using personalized, optimized high-definition montages are needed in both Sz and HC groups. Second, there are many other potential nodes of relevance to the SRTT, such as striatum and cerebellum [e.g. [29, 47, 97]], that were not assessed.

Finally, all Sz participants were receiving antipsychotic medication, which may have affected results although no correlations with standardized dose were observed. Nevertheless, a definitive test of the role of antipsychotics would require determination of antipsychotic blood levels, which were not performed. A subset of Sz participants were also receiving anticholinergic medications. Use of medications may contribute to between-group differences. In general, D2 agonists have been shown to enhance effects of tDCS-induced plasticity to both cathodal and anodal stimulation, whereas D2 antagonists reduce plasticity [98, 99] although inverted U-shaped curves have also been described [100, 101]. Thus, it is unlikely that medication status is responsible for the differential effects of tDCS by polarity and location, although further effects might have been observed in the absence of medication. Although unblinding due to scalp sensation is always a potential concern in tDCS studies, the scalp sensation elicited by active vs. sham tDCS did not differ significantly by group or condition. Moreover, it is unlikely that unblinding would have resulted in the specific patterns of physiological alteration observed in this study across tDCS conditions.

## Conclusions

In summary, motor learning is a fundamental skill to our daily lives [102]. Motor performance and its dysfunction in Sz has been associated with poor social and functional outcomes and contributes to decreased quality of life, impaired work capacity, and a reduced life expectancy by 10-20 years [103]. Here, we demonstrate that the SRTT combined with source-space EEG analysis may be used both as a method for investigating mechanisms of motor and procedural learning deficits in Sz, and as a mechanism to develop refined non-invasive brain stimulation approaches for modulation of ongoing functional connectivity impairments across relevant disorders.

### Supplementary information

Supplementary methods

## References

[1] Green MF, Horan WP, Lee J (2019). Nonsocial and social cognition in schizophrenia: current evidence and future directions. World Psychiatry.

[2] Javitt DC (2023). Cognitive impairment associated with schizophrenia: from pathophysiology to treatment. Annu Rev Pharm Toxicol.

[3] Polania R, Nitsche MA, Ruff CC (2018). Studying and modifying brain function with non-invasive brain stimulation. Nat Neurosci.

[4] Boroda E, Sponheim SR, Fiecas M, Lim KO (2020). Transcranial direct current stimulation (tDCS) elicits stimulus-specific enhancement of cortical plasticity. Neuroimage.

[5] Focke J, Kemmet S, Krause V, Keitel A, Pollok B (2017). Cathodal transcranial direct current stimulation (tDCS) applied to the left premotor cortex (PMC) stabilizes a newly learned motor sequence. Behav Brain Res.

[6] Ghanavati E, Salehinejad MA, De Melo L, Nitsche MA, Kuo MF (2022). NMDA receptor-related mechanisms of dopaminergic modulation of tDCS-induced neuroplasticity. Cereb Cortex.

[7] Brunoni AR, Shiozawa P, Truong D, Javitt DC, Elkis H, Fregni F (2014). Understanding tDCS effects in schizophrenia: a systematic review of clinical data and an integrated computation modeling analysis. Expert Rev Med devices.

[8] Stuchlikova Z, Klirova M (2022). A literature mini-review of transcranial direct current stimulation in schizophrenia. Front Psychiatry.

[9] Rosson S, de Filippis R, Croatto G, Collantoni E, Pallottino S, Guinart D (2022). Brain stimulation and other biological non-pharmacological interventions in mental disorders: An umbrella review. Neurosci Biobehav Rev.

[10] Hyde J, Carr H, Kelley N, Seneviratne R, Reed C, Parlatini V (2022). Efficacy of neurostimulation across mental disorders: systematic review and meta-analysis of 208 randomized controlled trials. Mol Psychiatry.

[11] Brunelin J, Adam O, Mondino M (2022). Recent advances in noninvasive brain stimulation for schizophrenia. Curr Opin Psychiatry.

[12] Yamada Y, Sumiyoshi T (2021). Neurobiological mechanisms of transcranial direct current stimulation for psychiatric disorders; neurophysiological, chemical, and anatomical considerations. Front Hum Neurosci.

[13] Fregni F, El-Hagrassy MM, Pacheco-Barrios K, Carvalho S, Leite J, Simis M (2021). Evidence-based guidelines and secondary meta-analysis for the use of transcranial direct current stimulation in neurological and psychiatric disorders. Int J Neuropsychopharmacol.

[14] Ciullo V, Spalletta G, Caltagirone C, Banaj N, Vecchio D, Piras F (2021). Transcranial direct current stimulation and cognition in neuropsychiatric disorders: systematic review of the evidence and future directions. Neuroscientist.

[15] Buch ER, Santarnecchi E, Antal A, Born J, Celnik PA, Classen J (2017). Effects of tDCS on motor learning and memory formation: A consensus and critical position paper. Clin Neurophysiol.

[16] Savic B, Meier B (2016). How transcranial direct current stimulation can modulate implicit motor sequence learning and consolidation: a brief review. Front Hum Neurosci.

[17] Shilo G, Lavidor M (2019). Non-linear effects of cathodal transcranial direct current stimulation (tDCS) of the primary motor cortex on implicit motor learning. Exp Brain Res.

[18] Pergher V, Au J, Alizadeh Shalchy M, Santarnecchi E, Seitz A, Jaeggi SM (2022). The benefits of simultaneous tDCS and working memory training on transfer outcomes: A systematic review and meta-analysis. Brain Stimul.

[19] Clark GM, Lum JAG (2017). Procedural learning in Parkinson’s disease, specific language impairment, dyslexia, schizophrenia, developmental coordination disorder, and autism spectrum disorders: A second-order meta-analysis. Brain Cogn.

[20] Siegert RJ, Weatherall M, Bell EM (2008). Is implicit sequence learning impaired in schizophrenia? A meta-analysis. Brain Cogn.

[21] Nitsche MA, Schauenburg A, Lang N, Liebetanz D, Exner C, Paulus W (2003). Facilitation of implicit motor learning by weak transcranial direct current stimulation of the primary motor cortex in the human. J Cogn Neurosci.

[22] Lopez-Alonso V, Cheeran B, Fernandez-del-Olmo M (2015). Relationship between non-invasive brain stimulation-induced plasticity and capacity for motor learning. Brain Stimul.

[23] Rivera-Urbina GN, Molero-Chamizo A, Nitsche MA (2022). Discernible effects of tDCS over the primary motor and posterior parietal cortex on different stages of motor learning. Brain Struct Funct.

[24] Donders FC (1969). On the speed of mental processes. Acta Psychol (Amst).

[25] Sehatpour P, Donde C, Hoptman MJ, Kreither J, Adair D, Dias E (2020). Network-level mechanisms underlying effects of transcranial direct current stimulation (tDCS) on visuomotor learning. Neuroimage.

[26] Sehatpour P, Donde C, Adair D, Kreither J, Lopez-Calderon J, Avissar M (2021). Comparison of cortical network effects of high-definition and conventional tDCS during visuomotor processing. Brain Stimul.

[27] Nuechterlein KH, Green MF, Kern RS, Baade LE, Barch DM, Cohen JD (2008). The MATRICS Consensus Cognitive Battery, part 1: test selection, reliability, and validity. Am J psychiatry.

[28] Ashe J, Lungu OV, Basford AT, Lu X (2006). Cortical control of motor sequences. Curr Opin Neurobiol.

[29] Hardwick RM, Rottschy C, Miall RC, Eickhoff SB (2013). A quantitative meta-analysis and review of motor learning in the human brain. Neuroimage.

[30] Kantak SS, Mummidisetty CK, Stinear JW (2012). Primary motor and premotor cortex in implicit sequence learning–evidence for competition between implicit and explicit human motor memory systems. Eur J Neurosci.

[31] Grafton ST, Hazeltine E, Ivry RB (1998). Abstract and effector-specific representations of motor sequences identified with PET. J Neurosci.

[32] Keele SW, Ivry R, Mayr U, Hazeltine E, Heuer H (2003). The cognitive and neural architecture of sequence representation. Psychol Rev.

[33] Gavornik JP, Bear MF (2014). Learned spatiotemporal sequence recognition and prediction in primary visual cortex. Nat Neurosci.

[34] Gallivan JP, Goodale MA (2018). The dorsal “action” pathway. Handb Clin Neurol.

[35] Causby R, Reed L, McDonnell M, Hillier S (2014). Use of objective psychomotor tests in health professionals. Percept Mot Skills.

[36] Sigirtmac IC, Oksuz C (2022). Determination of the optimal cutoff values and validity of the Purdue Pegboard Test. Br J Occup Ther.

[37] Pfurtscheller G, Lopes da Silva FH (1999). Event-related EEG/MEG synchronization and desynchronization: basic principles. Clin Neurophysiol.

[38] Roelfsema PR, Engel AK, Konig P, Singer W (1997). Visuomotor integration is associated with zero time-lag synchronization among cortical areas. Nature.

[39] Gompf F, Pflug A, Laufs H, Kell CA (2017). Non-linear relationship between BOLD activation and amplitude of beta oscillations in the supplementary motor area during rhythmic finger tapping and internal timing. Front Hum Neurosci.

[40] Gladwin TE, t Hart BM, de Jong R (2008). Dissociations between motor-related EEG measures in a cued movement sequence task. Cortex.

[41] Jasper H, Penfield W (1949). Electrocorticograms in man: Effect of voluntary movement upon the electrical activity of the precentral gyrus. Arch für Psychiatr und Nervenkrankheiten.

[42] Neuper C, Pfurtscheller G (2001). Evidence for distinct beta resonance frequencies in human EEG related to specific sensorimotor cortical areas. Clin Neurophysiol.

[43] Khanna P, Carmena JM (2015). Neural oscillations: beta band activity across motor networks. Curr Opin Neurobiol.

[44] Weinrich CA, Brittain JS, Nowak M, Salimi-Khorshidi R, Brown P, Stagg CJ (2017). Modulation of long-range connectivity patterns via frequency-specific stimulation of human cortex. Curr Biol.

[45] Brovelli A, Ding M, Ledberg A, Chen Y, Nakamura R, Bressler SL (2004). Beta oscillations in a large-scale sensorimotor cortical network: directional influences revealed by Granger causality. Proc Natl Acad Sci USA.

[46] Schoffelen JM, Oostenveld R, Fries P (2008). Imaging the human motor system’s beta-band synchronization during isometric contraction. Neuroimage.

[47] Tzvi E, Munte TF, Kramer UM (2014). Delineating the cortico-striatal-cerebellar network in implicit motor sequence learning. Neuroimage.

[48] Mondino M, Sauvanaud F, Brunelin J (2018). A review of the effects of transcranial direct current stimulation for the treatment of hallucinations in patients with schizophrenia. J ECT.

[49] Jiang WL, Cai DB, Sun CH, Yin F, Goerigk S, Brunoni AR (2022). Adjunctive tDCS for treatment-refractory auditory hallucinations in schizophrenia: A meta-analysis of randomized, double-blinded, sham-controlled studies. Asian J Psychiatr.

[50] Adam O, Blay M, Brunoni AR, Chang HA, Gomes JS, Javitt DC (2022). Efficacy of transcranial direct current stimulation to improve insight in patients with schizophrenia: a systematic review and meta-analysis of randomized controlled trials. Schizophr Bull.

[51] Frase L, Mertens L, Krahl A, Bhatia K, Feige B, Heinrich SP (2021). Transcranial direct current stimulation induces long-term potentiation-like plasticity in the human visual cortex. Transl Psychiatry.

[52] Jahshan C, Wynn JK, Roach BJ, Mathalon DH, Green MF (2020). Effects of transcranial direct current stimulation on visual neuroplasticity in schizophrenia. Clin EEG Neurosci.

[53] Sehatpour P, Bassir Nia A, Adair D, Wang Z, DeBaun HM, Silipo G (2020). Multimodal computational modeling of visual object recognition deficits but intact repetition priming in schizophrenia. Front Psychiatry.

[54] Amiaz R, Vainiger D, Gershon AA, Weiser M, Lavidor M, Javitt DC (2016). Applying Transcranial Magnetic Stimulation (TMS) Over the dorsal visual pathway induces schizophrenia-like disruption of perceptual closure. Brain Topogr.

[55] Martinez A, Tobe R, Dias EC, Ardekani BA, Veenstra-VanderWeele J, Patel G (2019). Differential patterns of visual sensory alteration underlying face emotion recognition impairment and motion perception deficits in schizophrenia and autism spectrum disorder. Biol psychiatry.

[56] Pobric G, Hulleman J, Lavidor M, Silipo G, Rohrig S, Dias E (2018). Seeing the world as it is: mimicking veridical motion perception in schizophrenia using non-invasive brain stimulation in healthy participants. Brain Topogr.

[57] King JP, Christensen BK, Westwood DA (2008). Grasping behavior in schizophrenia suggests selective impairment in the dorsal visual pathway. J Abnorm Psychol.

[58] Sanfratello L, Aine C, Stephen J (2018). Neuroimaging investigations of dorsal stream processing and effects of stimulus synchrony in schizophrenia. Psychiatry Res Neuroimaging.

[59] Cadenhead KS, Serper Y, Braff DL (1998). Transient versus sustained visual channels in the visual backward masking deficits of schizophrenia patients. Biol psychiatry.

[60] Ammons RB, Ammons CH (1962). The Quick Test (QT): provisional manual. Psychol Rep.

[61] Tiffin J, Asher EJ (1948). The Purdue pegboard; norms and studies of reliability and validity. J Appl Psychol.

[62] Wang YC, Magasi SR, Bohannon RW, Reuben DB, McCreath HE, Bubela DJ (2011). Assessing dexterity function: a comparison of two alternatives for the NIH Toolbox. J Hand Ther.

[63] Revheim N, Medalia A (2004). The independent living scales as a measure of functional outcome for schizophrenia. Psychiatr Serv.

[64] Nitsche MA, Paulus W (2001). Sustained excitability elevations induced by transcranial DC motor cortex stimulation in humans. Neurology.

[65] Zhao H, Qiao L, Fan D, Zhang S, Turel O, Li Y (2017). Modulation of brain activity with noninvasive Transcranial Direct Current Stimulation (tDCS): Clinical applications and safety concerns. Front Psychol.

[66] Huang Y, Datta A, Bikson M, Parra LC (2018). ROAST: An open-source, fully-automated, realistic volumetric-approach-based simulator For TES. Annu Int Conf IEEE Eng Med Biol Soc.

[67] Hockenberry MJ, Wilson D, Winkelstein ML. Wong’s Essentials of Pediatric Nursing. 7 edn. Mosby: St Louis, 2005, p 1259.

[68] Delorme A, Makeig S (2004). EEGLAB: an open source toolbox for analysis of single-trial EEG dynamics including independent component analysis. J Neurosci methods.

[69] Lopez-Calderon J, Luck SJ (2014). ERPLAB: an open-source toolbox for the analysis of event-related potentials. Front Hum Neurosci.

[70] Perrin F, Pernier J, Bertrand O, Giard MH, Echallier JF (1987). Mapping of scalp potentials by surface spline interpolation. Electroencephalogr Clin Neurophysiol.

[71] Hoechstetter K, Bornfleth H, Weckesser D, Ille N, Berg P, Scherg M (2004). BESA source coherence: a new method to study cortical oscillatory coupling. Brain Topogr.

[72] Sehatpour P, Molholm S, Schwartz TH, Mahoney JR, Mehta AD, Javitt DC (2008). A human intracranial study of long-range oscillatory coherence across a frontal-occipital-hippocampal brain network during visual object processing. Proc Natl Acad Sci USA.

[73] Van Veen BD, van Drongelen W, Yuchtman M, Suzuki A (1997). Localization of brain electrical activity via linearly constrained minimum variance spatial filtering. IEEE Trans Biomed Eng.

[74] Sekihara K, Nagarajan SS, Poeppel D, Marantz A, Miyashita Y (2001). Reconstructing spatio-temporal activities of neural sources using an MEG vector beamformer technique. IEEE Trans Biomed Eng.

[75] Cohen MX *Analyzing Neural Time Series Data: Theory and Practice*, 10.7551/mitpress/9609.001.0001. The MIT Press2014.

[76] Yeo BT, Krienen FM, Sepulcre J, Sabuncu MR, Lashkari D, Hollinshead M (2011). The organization of the human cerebral cortex estimated by intrinsic functional connectivity. J Neurophysiol.

[77] Kuo MF, Chen PS, Nitsche MA (2017). The application of tDCS for the treatment of psychiatric diseases. Int Rev Psychiatry.

[78] Garvey MA, Cuthbert BN (2017). Developing a motor systems domain for the NIMH RDoC Program. Schizophr Bull.

[79] Mittal VA, Bernard JA, Northoff G (2017). What can different motor circuits tell us about psychosis? An RDoC perspective. Schizophr Bull.

[80] Dickson H, Roberts RE, To M, Wild K, Loh M, Laurens KR (2020). Adolescent trajectories of fine motor and coordination skills and risk for schizophrenia. Schizophr Res.

[81] Hidese S, Ota M, Sasayama D, Matsuo J, Ishida I, Hiraishi M (2018). Manual dexterity and brain structure in patients with schizophrenia: A whole-brain magnetic resonance imaging study. Psychiatry Res Neuroimaging.

[82] Sehatpour P, Molholm S, Javitt DC, Foxe JJ (2006). Spatiotemporal dynamics of human object recognition processing: an integrated high-density electrical mapping and functional imaging study of “closure” processes. Neuroimage.

[83] Dias EC, Sheridan H, Martinez A, Sehatpour P, Silipo G, Rohrig S (2021). Neurophysiological, oculomotor, and computational modeling of impaired reading ability in schizophrenia. Schizophr Bull.

[84] Revheim N, Corcoran CM, Dias E, Hellmann E, Martinez A, Butler PD (2014). Reading deficits in schizophrenia and individuals at high clinical risk: relationship to sensory function, course of illness, and psychosocial outcome. Am J Psychiatry.

[85] Martinez A, Revheim N, Butler PD, Guilfoyle DN, Dias EC, Javitt DC (2012). Impaired magnocellular/dorsal stream activation predicts impaired reading ability in schizophrenia. Neuroimage Clin.

[86] Bikson M, Brunoni AR, Charvet LE, Clark VP, Cohen LG, Deng ZD (2018). Rigor and reproducibility in research with transcranial electrical stimulation: An NIMH-sponsored workshop. Brain Stimul.

[87] Lopes JBP, Miziara IM, Kahani D, Cordeiro LB, Fonseca PR, Lazzari RD (2022). Electroencephalographic analysis of brain activity after interventions with transcranial direct current stimulation over the motor cortex: a systematic review. Adapt Behav.

[88] Uhlhaas PJ, Singer W (2006). Neural synchrony in brain disorders: relevance for cognitive dysfunctions and pathophysiology. Neuron.

[89] Uhlhaas PJ, Haenschel C, Nikolic D, Singer W (2008). The role of oscillations and synchrony in cortical networks and their putative relevance for the pathophysiology of schizophrenia. Schizophr Bull.

[90] Peter J, Ferraioli F, Mathew D, George S, Chan C, Alalade T (2022). Movement-related beta ERD and ERS abnormalities in neuropsychiatric disorders. Front Neurosci.

[91] Gascoyne LE, Brookes MJ, Rathnaiah M, Katshu M, Koelewijn L, Williams G (2021). Motor-related oscillatory activity in schizophrenia according to phase of illness and clinical symptom severity. Neuroimage Clin.

[92] Rathnaiah M, Liddle EB, Gascoyne L, Kumar J, Zia Ul Haq Katshu M, Faruqi C (2020). Quantifying the core deficit in classical schizophrenia. Schizophr Bull Open.

[93] Hoptman MJ, Parker EM, Nair-Collins S, Dias EC, Ross ME, DiCostanzo JN (2018). Sensory and cross-network contributions to response inhibition in patients with schizophrenia. Neuroimage Clin.

[94] Hoptman MJ, Tural U, Lim KO, Javitt DC, Oberlin LE (2022). Relationships between Diffusion Tensor Imaging and Resting State Functional Connectivity in Patients with Schizophrenia and Healthy Controls: A Preliminary Study. Brain Sci.

[95] Hearne LJ, Mill RD, Keane BP, Repovs G, Anticevic A, Cole MW. Activity flow underlying abnormalities in brain activations and cognition in schizophrenia. Sci Adv. 2021;7. 10.1126/sciadv.abf2513 PMC8279516 34261649

[96] Simpson MW, Mak M (2022). Single session transcranial direct current stimulation to the primary motor cortex fails to enhance early motor sequence learning in Parkinson’s disease. Behav Brain Res.

[97] Jongkees BJ, Immink MA, Boer OD, Yavari F, Nitsche MA, Colzato LS (2019). The effect of cerebellar tDCS on sequential motor response selection. Cerebellum.

[98] Kuo MF, Unger M, Liebetanz D, Lang N, Tergau F, Paulus W (2008). Limited impact of homeostatic plasticity on motor learning in humans. Neuropsychologia.

[99] Monte-Silva K, Liebetanz D, Grundey J, Paulus W, Nitsche MA (2010). Dosage-dependent non-linear effect of L-dopa on human motor cortex plasticity. J Physiol.

[100] Monte-Silva K, Kuo MF, Thirugnanasambandam N, Liebetanz D, Paulus W, Nitsche MA (2009). Dose-dependent inverted U-shaped effect of dopamine (D2-like) receptor activation on focal and nonfocal plasticity in humans. J Neurosci.

[101] Fresnoza S, Stiksrud E, Klinker F, Liebetanz D, Paulus W, Kuo MF (2014). Dosage-dependent effect of dopamine D2 receptor activation on motor cortex plasticity in humans. J Neurosci.

[102] Debas K, Carrier J, Orban P, Barakat M, Lungu O, Vandewalle G (2010). Brain plasticity related to the consolidation of motor sequence learning and motor adaptation. Proc Natl Acad Sci USA.

[103] Nadesalingam N, Chapellier V, Lefebvre S, Pavlidou A, Stegmayer K, Alexaki D (2022). Motor abnormalities are associated with poor social and functional outcomes in schizophrenia. Compr Psychiatry.

[104] Manoach DS, Cain MS, Vangel MG, Khurana A, Goff DC, Stickgold R (2004). A failure of sleep-dependent procedural learning in chronic, medicated schizophrenia. Biol Psychiatry.

