# Supplementary material for: Network-level mechanisms underlying effects of transcranial direct current stimulation (tDCS) on visuomotor learning in schizophrenia
Source: Transl Psychiatry. 2023 Nov 23;13:360. doi: 10.1038/s41398-023-02656-3 (PMC10665365; doi:10.1038/s41398-023-02656-3)
Supplement: Supplementary file 1 — Supplementary methods [file 41398_2023_2656_MOESM1_ESM.docx]

**Supplementary Methods**

tDCS Safety: We used the Wong-Baker Faces Pain Scale ^1^ to measure discomfort caused by tDCS application after each session. No pain-related adverse events occurred during the study.

*Single-trial behavioral analyses*: For single-trial analyses, single-trial log-RT distributions were compared across conditions using single vs. dual-Gaussian models using GraphPad 7.0 non-linear curve fitting functions. For each analysis, both single Guassian and dual Gaussian fits were considered. For the single Guassian model, data were fit to the formula (# of responses @ log-RT=X) = (Total # of responses across RTs) * e^-(X-Mean/SD^2)^, where Total number of responses, Mean, and SD were modeled parameters. All parameters were constrained to be positive values. For the dual Gaussian model, a second set of parameters corresponding to the second Gaussian distribution were added. Starting values were provided based upon apparent peaks in the histogram plots.

In all cases, the simpler model (single Gaussian) was chosen unless the more complex model (Dual Gaussian) was shown to be statistically superior. Comparison between models was assessed using a goodness-of-fit ANOVA. In addition, absolute goodness of fit (R^2^) was required to be >95% for all accepted fits. Initial analyses were performed using data from the random repeat runs only, which yielded unimodal models in all cases. Mean RT values from the random runs were used to constrain the slow RT component for the subsequent analyses of RT data from the fixed-sequence blocks. Initial values for each model were provided based upon visual inspection of RT histograms. Analyses were conducted both by quarter to evaluate stability of RTs over the course of the training and collapsed across quarters to compare coefficients.

Comparison of %age fast responses across tDCS conditions was performed by comparing dual Gaussian models in which the ratio between fast and slow responses was assumed to be constant vs. those in which it was assumed to vary across conditions. The simpler model (all %ages equal) was accepted unless the more complex model (%ages different) was found to be statistically superior.

*EEG artifact removal*: The recording of EEG during tDCS requires particular attention because neurostimulation may also affect electrophysiological measurement ^2^. These include cardiac and ocular motor distortion, as inherent, and motion and myogenic distortion as non-inherent physiological artifacts ^2^. Prior studies, including ours, have successfully removed EEG artifacts caused by concurrent tDCS ^3-5^.

*Beamformer:* Our prior tDCS studies of the cortical network involved in SRTT ^4,5^, indicated the modulatory effects of tDCS within a time window of 100 ms prior to the motor response in the β frequency range 12Hz -24HZ. β-ERD values were calculated using temporal spectral evolution (TSE) defined as the relative power change at a time-frequency bin compared with the mean power over the baseline epoch for that frequency ^6,7^. Intracranial sources of beta-activity were assessed using a Beamformer approach ^5,8,9^, which involves the following steps: 1) For each channel single-trial data in time domain is transformed into time-frequency domain in order to compute the complex time-frequency signal ^6^; 2) Complex cross-spectral density matrices is then computed for each trial; 3) A forward model is applied, and a lead-field matrix is estimated; and 4) The brain in Talairach space is divided into a grid and the normalized task-dependent contribution (q) to a given time-frequency range of interest from every location on this grid is then estimated.

Each channel single-trial data in time domain is transformed into time-frequency domain, deriving $S_{x,n}(f,t)=A_{x,n}(f,t)\cdot e^{i\phi_{x,n}(f,t)}$ as the complex time-frequency signal of channel x in trial n at frequency f and latency t, characterized by its amplitude A and its phase φ.

Complex cross spectral density matrices C are then computed for each trial:

$C_{xy}(f,t)=S_{x,n}(f,t)\cdot{S^{*}}_{y,n}(f,t)$ Here, ^*^ indicates the complex conjugate.

The output power P of the beamformer for a specific brain region at location r is then computed as ^10^: $P\left( r \right)=tr^{'\left[ L^{T}\left( r \right).C_{r}^{-1}.L\left( r \right) \right]^{-1}}$

Here $C_{r}^{-1}$ is the inverse of the average of $C_{xy}\left( f,t \right)$ over trials and the time-frequency range of interest, L is the leadfield matrix (i.e. the magnitude of the signal each source contributes to each recording sensor ^11^ of the model containing the regional source ^12,13^ at location r. In computing the lead-field matrix we used the standardized finite element model (FEM) implemented in BESA. The FEM model provides a realistic approximation to the averaged head and uses three compartments: brain/CSF, skull and scalp to describes the electrical conductivity distribution inside of the head. T is the matrix transpose and tr’[] is the trace of the [3x3] submatrix (of the expression in the bracket) for the source at location r.

We then normalized the power P(r) with the power at the corresponding time-frequency interval of the baseline P_baseline_(r) to obtain the value q(r):

$q\left( r \right)=\left\{ \begin{aligned} \sqrt{\frac{P\left( r \right)}{P_{baseline}\left( r \right)}}-1 for P\left( r \right)\geq P_{baseline}\left( r \right) \\ 1-\sqrt{\frac{P_{baseline}\left( r \right)}{P\left( r \right)}} for P(r)<P_{baseline}(r) \end{aligned} \right.$

The brain in Talairach space is divided into a grid with a resolution of 5 mm^3^ and the beamformer image is constructed from values q(r) computed from every location on this grid. q values are then shown in % where q[%] = q*100. This image is then extrapolated to a resolution of 1 mm^3^ and projected to an inflated brain image derived from an MRI of equal resolution. Since in the computation of beamformer image regional sources having three orthogonal vectors (i.e. radial, tangential and oblique) are used, projection onto an inflated brain surface more accurately represents the spread of the cortical activation. The overlap areas of the cortical projections obtained in HC and SZ groups are then used to determine the cortical regions with the highest q value which are then seeded with a virtual source ^14,15^, revealing three distinct cortical regions at MNI coordinates: PMC/SMA [-26, -10, 74], Motor [-40, -22, 60], Visual [-48, -78, 2]. The model was fitted to each individual and the goodness-of-fit (GOF) of the model was measured per individual. Hence, we were able to measure the SE of the GOF across the participants. The mean GOF value of the model in controls is 90.0 SE ± 2.0 and in Sz individuals is 85.0 SE ± 2.0.

In the next step single-trial source TF data was used to derive coherence measures across this cortical network as a measure of functional connectivity ^16-18^.

${C'}_{xy}(f,t)=\frac{\left| \sum_{n} S_{x,n}(f,t)\cdot{S^{*}}_{y,n}(f,t) \right|^{2}}{\sum_{n} \left| S_{x,n}(f,t) \right|^{2}\cdot\sum_{n} \left| S_{y,n}(f,t) \right|^{2}}$

Coherence ranges from 0 (no coherence) to 1 (maximum coherence). To determine the probability that coherence at a particular time-frequency sampling point is significantly higher than what is expected from random fluctuations is investigated based on an approach suggested by ^19^ and previously implemented and described by our group ^6^.

To investigate the probability that the coherence in sham differed significantly from the coherence in each of the other conditions, the individual subject mean coherence estimates were then subjected to a permutation cluster analysis ^4,20,21^. This approach is carried out in two general steps.

In the first step a Student’s paired t-test is carried out for every time-frequency (TF) bin to determine if there is a significant difference between the two conditions in the group. Here a cluster alpha level of 0.05 is set which allows us to identify the TF bins whose t-values exceed the 95^th^ quantile threshold and can be considered as a candidate to be included in a cluster of TF bins, which in our case are based on temporal and spectral contiguity. A cluster value is obtained by summing the t-values of the individual data bins in a cluster. This value serves as a test statistic for the next step of the analysis.

In the second step of the analysis, the clusters obtained in the preliminary parametric step are then submitted to permutation testing wherein the coherence data for sham gets systematically interchanged with the coherence data of the test condition. For each permutation, a new t-test is obtained per TF bin and a new test statistic (cluster-level summed t-values) is computed.

Here we have used 2000 permutations (drawn randomly without repetitions) from all possible permutations, i.e., 217. We used a meta-permutation test method proposed in ^22^. This method involves running the permutation testing many times (in our case 10 times) and averaging the results to obtain a final result. This approach is thought to be more stable than increasing the number of permutations. From the distribution of the test statistics obtained from our permutations we then calculate the proportion of the test values that are larger than the value obtained from the initial cluster obtained in step 1. Hence if less than 5% of all values are larger than the initial test value it is assumed that the data of the two conditions are not interchangeable with a chance level greater than 95% i.e. (P<.05).

**References**

1. Hockenberry M.J., Wilson D., Winkelstein M.L. Wong's Essentials of Pediatric Nursing. 7 ed. St Louis,: Mosby; 2005. p. 1259.

2. Gebodh N, Esmaeilpour Z, Adair D, . . . Bikson M. Inherent physiological artifacts in EEG during tDCS. Neuroimage. 2019;185:408-24. DOI: 10.1016/j.neuroimage.2018.10.025

3. Roy A, Baxter B, He B. High-definition transcranial direct current stimulation induces both acute and persistent changes in broadband cortical synchronization: a simultaneous tDCS-EEG study. IEEE Trans Biomed Eng. 2014;61(7):1967-78. DOI: 10.1109/TBME.2014.2311071

4. Sehatpour P, Donde C, Adair D, . . . Javitt DC. Comparison of cortical network effects of high-definition and conventional tDCS during visuomotor processing. Brain Stimul. 2021;14(1):33-5. DOI: 10.1016/j.brs.2020.11.004

5. Sehatpour P, Donde C, Hoptman MJ, . . . Javitt DC. Network-level mechanisms underlying effects of transcranial direct current stimulation (tDCS) on visuomotor learning. Neuroimage. 2020;223:117311. DOI: 10.1016/j.neuroimage.2020.117311

6. Sehatpour P, Molholm S, Schwartz TH, . . . Foxe JJ. A human intracranial study of long-range oscillatory coherence across a frontal-occipital-hippocampal brain network during visual object processing. Proc Natl Acad Sci U S A. 2008;105(11):4399-404. DOI: 10.1073/pnas.0708418105

7. Pfurtscheller G, Lopes da Silva FH. Event-related EEG/MEG synchronization and desynchronization: basic principles. Clin Neurophysiol. 1999;110(11):1842-57. DOI: 10.1016/s1388-2457(99)00141-8

8. Van Veen BD, van Drongelen W, Yuchtman M, Suzuki A. Localization of brain electrical activity via linearly constrained minimum variance spatial filtering. IEEE Trans Biomed Eng. 1997;44(9):867-80. DOI: 10.1109/10.623056

9. Sekihara K, Nagarajan SS, Poeppel D, Marantz A, Miyashita Y. Reconstructing spatio-temporal activities of neural sources using an MEG vector beamformer technique. IEEE Trans Biomed Eng. 2001;48(7):760-71. DOI: 10.1109/10.930901

10. Gross J, Kujala J, Hamalainen M, . . . Salmelin R. Dynamic imaging of coherent sources: Studying neural interactions in the human brain. Proc Natl Acad Sci U S A. 2001;98(2):694-9. DOI: 10.1073/pnas.98.2.694

11. Scherg M, von Cramon D. Two bilateral sources of the late AEP as identified by a spatiotemporal dipole model. Electroenceph clin Neurophysiol. 1985;62:32-44.

12. Scherg M, Picton TW. Separation and identification of event-related potential components by brain electric source analysis. Electroencephalogr Clin Neurophysiol Suppl. 1991;42:24-37.

13. Sehatpour P, Molholm S, Javitt DC, Foxe JJ. Spatiotemporal dynamics of human object recognition processing: an integrated high-density electrical mapping and functional imaging study of "closure" processes. Neuroimage. 2006;29(2):605-18. DOI: 10.1016/j.neuroimage.2005.07.049

14. Hoechstetter K, Bornfleth H, Weckesser D, . . . Scherg M. BESA source coherence: a new method to study cortical oscillatory coupling. Brain Topogr. 2004;16(4):233-8. DOI: 10.1023/b:brat.0000032857.55223.5d

15. Scherg M, Berg P, Nakasato N, Beniczky S. Taking the EEG Back Into the Brain: The Power of Multiple Discrete Sources. Front Neurol. 2019;10:855. DOI: 10.3389/fneur.2019.00855

16. Bressler SL. Large-scale cortical networks and cognition. Brain Res Brain Res Rev. 1995;20(3):288-304. DOI: 10.1016/0165-0173(94)00016-i

17. Bressler SL, Coppola R, Nakamura R. Episodic multiregional cortical coherence at multiple frequencies during visual task performance. Nature. 1993;366(6451):153-6. DOI: 10.1038/366153a0

18. Fries P. A mechanism for cognitive dynamics: neuronal communication through neuronal coherence. Trends in cognitive sciences. 2005;9(10):474-80. DOI: 10.1016/j.tics.2005.08.011

19. Lachaux JP, Rodriguez E, Martinerie J, Varela FJ. Measuring phase synchrony in brain signals. Hum Brain Mapp. 1999;8(4):194-208. DOI: 10.1002/(sici)1097-0193(1999)8:4<194::aid-hbm4>3.0.co;2-c

20. Bullmore ET, Suckling J, Overmeyer S, . . . Brammer MJ. Global, voxel, and cluster tests, by theory and permutation, for a difference between two groups of structural MR images of the brain. IEEE Trans Med Imaging. 1999;18(1):32-42. DOI: 10.1109/42.750253

21. Maris E, Oostenveld R. Nonparametric statistical testing of EEG- and MEG-data. Journal of neuroscience methods. 2007;164(1):177-90. DOI: 10.1016/j.jneumeth.2007.03.024

22. Cohen MX. Analyzing Neural Time Series Data: Theory and Practice: The MIT Press; 2014.
